# Evaluation of hyperspectral LiDAR for monitoring rice leaf nitrogen by comparison with multispectral LiDAR and passive spectrometer

**DOI:** 10.1038/srep40362

**Published:** 2017-01-16

**Authors:** Jia Sun, Shuo Shi, Wei Gong, Jian Yang, Lin Du, Shalei Song, Biwu Chen, Zhenbing Zhang

**Affiliations:** 1 State Key Laboratory of Information Engineering in Surveying, Mapping and Remote Sensing, Wuhan University, Wuhan, Hubei 430079, China; 2 Collaborative Innovation Center of Geospatial Technology, Wuhan, Hubei 430079, China; 3 School of Physics and Technology, Wuhan University, Wuhan, Hubei 430072, China; 4 Wuhan Institute of Physics and Mathematics, Chinese Academy of Sciences, Wuhan, Hubei 430071, China

## Abstract

Fast and nondestructive assessment of leaf nitrogen concentration (LNC) is critical for crop growth diagnosis and nitrogen management guidance. In the last decade, multispectral LiDAR (MSL) systems have promoted developments in the earth and ecological sciences with the additional spectral information. With more wavelengths than MSL, the hyperspectral LiDAR (HSL) system provides greater possibilities for remote sensing crop physiological conditions. This study compared the performance of ASD FieldSpec Pro FR, MSL, and HSL for estimating rice (*Oryza sativa*) LNC. Spectral reflectance and biochemical composition were determined in rice leaves of different cultivars (Yongyou 4949 and Yangliangyou 6) throughout two growing seasons (2014–2015). Results demonstrated that HSL provided the best indicator for predicting rice LNC, yielding a coefficient of determination (*R*^*2*^) of 0.74 and a root mean square error of 2.80 mg/g with a support vector machine, similar to the performance of ASD (*R*^*2*^ = 0.73). Estimation of rice LNC could be significantly improved with the finer spectral resolution of HSL compared with MSL (*R*^*2*^ = 0.56).

Rice (*Oryza sativa*) is a daily necessity among people. The expansion of cities has resulted in a decrease in available land for paddies. In addition, immoderate application of fertilizers has given rise to serious environmental consequences, such as water eutrophication and soil hardening^1^. Nitrogenous fertilizer is used extensively because nitrogen (N) supply is a crucial factor in improving crop yields. Thus, accurate monitoring of the status of rice leaf N concentration (LNC) not only enables high yields but also improves the efficiency of applied nitrogenous fertilizer and prevents eutrophication. As an important indicator for crop growth diagnosis, the concept of plant N concentration is based on dry matter^2^, while plant N content is based on field area, and is the product of N concentration and dry biomass^3^. Considering that plant N content is strongly influenced by growth stages, we measured LNCs in the present study.

Multispectral and hyperspectral remote sensing are nondestructive methods of estimating the foliar biochemical concentration of vegetation^4^,^5^. This method has been used to monitor the chlorophyll, lignin, N, and water status of vegetation^3^,^6^,^7^. The influence of factors, including canopy structure, needs to be eliminated before using canopy reflectance to estimate N concentration^8^. However, passive remote sensing is often influenced by multiple aerial/atmospheric condition factors, including pollution, clouds, and solar zenith angle.

Given the advantage of obtaining precise three-dimensional (3D) information, light detection and ranging (LiDAR) has undergone advanced developments in quantifying the 3D surface properties and processes in earth and ecological sciences^9^,^10^. The potential use of the intensity of point clouds in plant biochemistry estimation needs further exploration^11^. LiDAR intensity is useful in retrieving plant chlorophyll content^12^, nitrogen status^13^, and leaf water content^14^. Compared with traditional single-wavelength LiDAR systems such as active flash sensor (AFS) and GreenSeeker (NTech Industries, Inc., Ukiah, CA), multispectral LiDAR (MSL) and hyperspectral LiDAR (HSL) with high spectral resolution allow for increased sensitivity to characterize leaf biochemistry by emitting separate laser beams simultaneously, or utilizing a supercontinuum laser source with a wide spectrum range and a multi-channel detector.

Recently, MSL/HSL systems have been applied to estimate leaf moisture contents under laboratory conditions^15^, represent the chlorophyll content of harvested Scots pine shoots^16^, and reflect leaf nitrogen content levels^17^. A few promising commercial multispectral laser scanners have been developed^18^,^19^,^20^. Nevertheless, their wavelength number is limited to 2–3. Moreover, different channels frequently do not follow the same optical path, which can result in a series of noises and errors.

With regard to pattern recognition algorithms, multiple linear regression methods such as partial least-squares regression^21^ have been widely applied to estimate vegetation biochemical parameters^22^,^23^. However, the exact relationship between spectral reflectance and LNC may not be linear. A decision tree is quick to train and execute, able to deal with non-linear relationships between features and classes and with other advantages. However, it can have difficulty handling too many features^17^. A support vector machine (SVM) is a popular machine learning method for data classification and regression; this method has been successfully applied in remote sensing^24^,^25^. Advantages of SVM include robustness, insensitivity to the number of dimensions, and small sample size requirement for training^26^.

The development of HSL offers a way to address the limitations of traditional optical remote sensing and LiDAR. However, the performance of HSL in quantifying LNC has yet to be directly compared with that of other remote sensing technologies. The current study compared the performance of an HSL system in discriminating rice LNC with that of the passive sensor ASD FieldSpec Pro FR (Analytical Spectral Devices, field spectroradiometer, full-range, Inc., Boulder, USA) and an MSL system using SVM. The experiments were based on rice samples of different cultivars and growth states, grown in different places for two consecutive years (2014–2015). The objectives of this study were to (1) estimate rice LNC in different situations (cultivars, growth stages, and different field locations) with data collected by three sensors (ASD, MSL, and HSL) and (2) compare their capability to predict rice LNC with SVM. This study focused mainly on the novel use of the LiDAR intensities of HSL and MSL to reflect foliar biochemistry.

## Materials and Methods

### Study sites and leaf sampling

**Figure 1:**
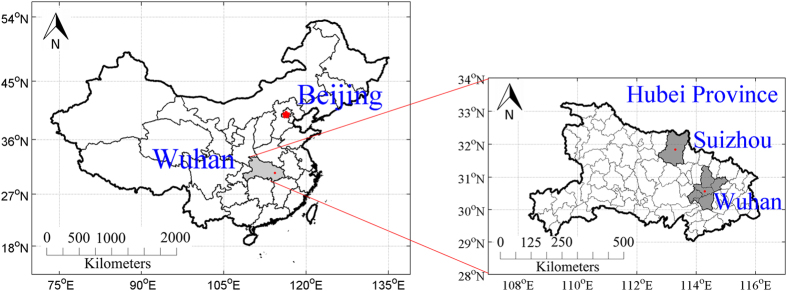
Location of study sites: location of Hubei Province in Greater China, and locations of Suizhou and Wuhan in Hubei Province [constructed by MATLAB (R2011b, Mathworks Inc., Natick, MA, USA)].

Located on the Jianghan Plain, Hubei Province is one of the largest provinces for rice plantations in China. The yield of Hubei Province ranked first in the nation’s yields in 2014^27^. The experiments were conducted at two locations in Hubei Province: fields located in Junchuan County, Suizhou (113°13′26.52″E, 31°39′0.94″N) and the experimental station of Huazhong Agricultural University in Wuhan (114°21′9.27″E, 30°28′34.10″N) (Fig. 1). Different fertilizer treatments were applied in the fields sampled to provide a wide range of nitrogen concentrations.

Yongyou 4949 was grown in Junchuan County, Suizhou during the growing season of 2014. The crops were seeded on April 27, and transplanted on June 1. Six levels of urea fertilizer (0, 189, 229.5, 270, 310.5, and 351 kg/ha) were implemented. For every urea fertilizer level, three fields with identical cultivation conditions were used as replicates. The fields were designed as randomized blocks. Each plot was separated completely from the others by setting plastic films on the ridges of adjacent fields to avoid water leakage, thereby ensuring the precise application of N. The paddy rice samples were collected on July 15 and August 1, 2014.

Yangliangyou 6 was grown in the experimental station of the Huazhong Agricultural University in Wuhan in the growing season of 2015. The crops were seeded on April 30, and transplanted on May 27. Four levels of urea fertilizer (0, 120, 180, and 240 kg/ha) were implemented. Replications and separation between fields were similar to the procedures used in 2014. The paddy rice samples were gathered on July 20, 22, 24, and 26, 2015.

In each experimental field, at least six fully expanded second leaves from the top were selected randomly. The fresh leaf samples were sealed in plastic bags, kept in ice chests, and then transported to the laboratory for spectral measurements^28^ by ASD, MSL, and HSL. All samples were sent immediately to Wuhan Academy of Agricultural Science and Technology, where the Kjeldahl method^29^ was utilized to determine the paddy rice LNC.

### Measurements based on active and passive sensors

#### ASD field spectroradiometer

The passive sensor ASD FieldSpec Pro FR (Analytical Spectral Devices, field spectroradiometer, full-range, Inc., Boulder, USA) was used for spectrum measurements. The measurement was performed as described by Song, *et al*.^28^. The light source was a 100 W halogen reflectorized lamp. Each sample was measured three times. All spectra were obtained at the nadir direction of the radiometer with a 25° field of view (FOV) (resultant FOV diameter of 0.9 cm). Leaf radiance was measured initially in the range of 400–1000 nm at 1.4 nm intervals and 1000–2500 nm at 2.2 nm intervals, and then resampled automatically at 1 nm resolution. We measured a standard reference panel (Spectralon, Labsphere, Inc., North Sutton, NH, USA, reflectance nearly 99%) at several times during data acquisition. We obtained the reflectance of the target by dividing the radiance intensity of the target by that of the white panel.

Previous rice LNC studies showed that the highest *R*^*2*^ for LNC is concentrated in the red-edge bands (700–760 nm) paired with the red-edge to near-infrared (NIR) bands (700–1100 nm), followed by blue to green bands (450–520 nm) paired with red-edge to NIR bands (740–1000 nm)^30^. Moreover, N and chlorophyll are closely related, because chlorophylls are major nitrogen-containing components of plants^31^. Sensitivity analysis of chlorophyll conducted from field experiments^32^,^33^ and the leaf radiative transfer model of PROSPECT^34^ indicated that chlorophyll influences reflectance within the visible (VIS) and red-edge domain. We selected the VIS and NIR spectra (400–1000 nm) for this analysis because these regions of ASD data have high signal to noise ratio and avoid the water absorption bands present at higher wavelengths. In consideration that leaf reflectance was utilized in this study, canopy structural influences were not considered^8^. This range also avoids the influence of water^35^.

#### MSL system

**Figure 2:**
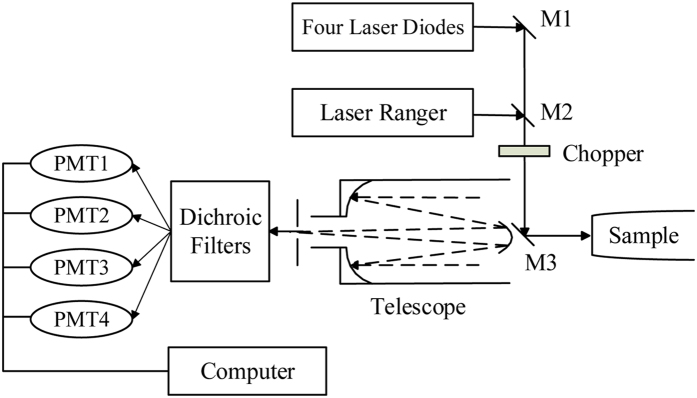
Optical layout of the employed multispectral LiDAR (MSL) system (PMT: photomultiplier).

One of the investigated active sensors was an MSL system developed by Wuhan University, operating at four wavelengths (556, 670, 700, and 780 nm) covering VIS and NIR wavelengths. A detailed description of the system is provided in the study by Wei, *et al*.^36^. The MSL system is composed of three parts: the laser emitting system, the receiver unit and the data-processing system (Fig. 2). Lasers are transmitted from four semiconductor laser diodes and synthesized into a single beam. After transferring to the detected leaf, the backscattered radiation is received by a Schmidt–Cassegrain telescope and detected by four photomultipliers. The connected computer then processes the acquired signals and LiDAR intensities. The MSL system functions on a motorized precision platform to ensure synchronous scanning and signal reception. In the experiment, all rice leaves were measured perpendicular at a distance of 3.7 m.

#### HSL system

**Figure 3:**
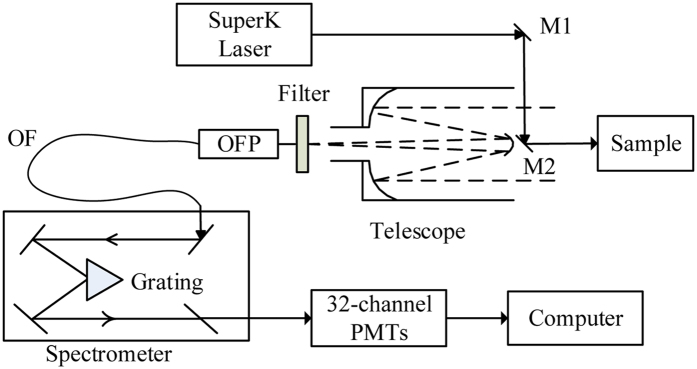
Optical layout of the employed hyperspectral LiDAR (HSL) system (OFP: optical fiber probe; OF: optical fiber; M1, M2: completely reflecting mirror; PMTs: photomultipliers).

The HSL system employed in this study was developed by Wuhan University, and a detailed description can be found in the study by Du, *et al*.^17^ (Fig. 3). A supercontinuum laser source was adopted to emit wide-band “white” laser. After the backscattered signals are collected by an achromatic telescope and collimated, the grating spectrometer (blazed grating) separates the maximum of single-slit diffraction from the zero-order maximum of multi-slit interference, thereby separating the echoes into different channels. With a multi-detector of 32 channels, the wavelength range of the HSL system is 538–910 nm. Finally, as a data acquisition detector, the photosensitive photomultiplier (PMT) arrays convert data to electronic signals.

All of the rice leaves were measured perpendicular at a distance of 4.2 m. Thus, the effects of incidence angle and distance were eliminated. The influence of certain factors, such as the dark current of the instrument, can be weakened by calculating the reflectance from the spectral measurements of a reference white panel^37^.

### Regression analysis

#### Data preprocessing

Foliar reflectance in the VIS and NIR regions (including the red edge) has often been considered a good candidate for representing the biochemical or biophysical parameters of vegetation^38^. On the basis of measurements of a standard white reference panel, a normalized laser return intensity can be obtained by dividing the raw laser return intensity value of the target with the averaged value of the panel. This normalized value is equivalent to the spectral reflectance in traditional optical remote sensing, and will be referred to as reflectance below. Three positions were randomly selected on each rice leaf sample and measured by the three sensors. The spectral values for all points per leaf were averaged. The collected spectral data were preprocessed in order to eliminate various random and environmental noises. The Savitzky–Golay smoothing filter^39^ with a third-order polynomial function and a bandwidth of 25 nm was applied to ASD data. In each MSL and HSL measurement, the spectra were collected for a point together with its 3D information. For the HSL spectrum, logarithmic and differential transformation^17^ were conducted. This procedure allows the analysis of the relationship between the leaf-level spectral characteristics in the VIS and NIR ranges as acquired by three detectors and laboratory-provided LNC (mass-based).

#### Support vector machine (SVM)

The exact relationship between LNC and the reflectance spectra remains unclear. With this consideration, a SVM, capable of constructing both linear and nonlinear inversion, was employed in this study. Different from an artificial neural network, a SVM has excellent generalization performance with a strong theoretical foundation in statistical learning theory^40^. SVM is insensitive to the number of dimensions and requires a small number of samples for training^26^.


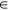
-support vector regression estimates an unknown continuous-valued function based on a finite number set of noisy samples. SVM regression performs linear regression in the high-dimension feature space by using insensitive loss and attempts to reduce model complexity by minimizing the empirical risk.

We consider a set of training points, 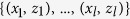, where 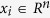 is a feature vector and 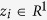 is the target output. Under given parameters C > 0 and 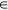
> 0, the standard form of SVM regression is:


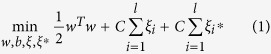


The dual problem is


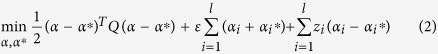


After solving problem (2), the approximate function is


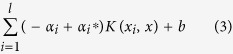


The model output α^*^−*α* is the result. Using MATLAB (R2011b, Mathworks Inc., Natick, MA, USA), where relevant inherent functions and the library LIBSVM^41^ are available, we used the algorithm of SVM to analyze the statistical relationship between the spectral reflectance acquired from different sensors and rice LNC. The radial basis function (RBF) was employed as a kernel function of SVM. The penalty parameters c and 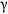 in RBF kernel were settled through five-fold cross validation, and these parameters differed in different regression models.

#### Statistical parameters

Altogether 220 rice samples were collected in 2014 and 2015. They were divided randomly into two datasets: 80% (176) as the training dataset and the remaining 20% (44) as the validation dataset for predicting LNC. The coefficient of determination (*R*^*2*^), root mean square error (*RMSE*), and relative error (*RE*) were calculated as shown below to evaluate the performance of the estimation models:


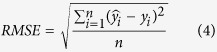



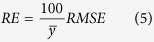


where ŷ_*i*_, *y*_*i*_, and 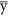 are the estimated, observed, and average observed rice LNC, respectively; *n* is the number ofsamples; and *RE* is the relative difference between the predicted and observed values.

## Results and Discussion

### Relationship between reflectance and rice LNC

**Figure 4:**
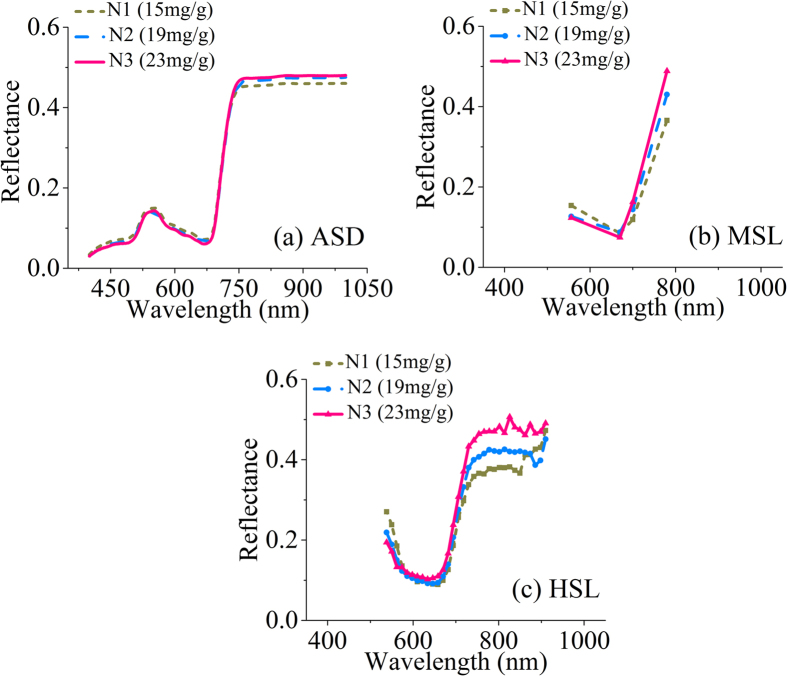
Rice leaf reflectance spectra under different leaf nitrogen concentration (LNC) levels and detected by different sensors (ASD: ASD FieldSpec Pro FR (Analytical Spectral Devices, field spectroradiometer, full-range, Inc., Boulder, USA); MSL: multispectral LiDAR; HSL: hyperspectral LiDAR; N1–N3 indicate different LNC levels).

The spectral reflectance of rice leaves was influenced strongly by the foliar chlorophyll concentration, which shares a close relationship to foliar N levels^2^,^17^,^31^. Therefore, observing leaf VIS and NIR reflective characteristics was a viable means to assess plant N concentration. The canopy structure, such as leaf area index (LAI) and canopy height, must be considered for green vegetation canopy reflectance^8^. Different LNC levels of rice can be approximately distinguished on the basis of the different spectral characteristics obtained using the investigated sensors (Fig. 4). The overall trend of the curves at the same N level was similar. In addition, the exact reflectance values detected by different sensors were not equal because of the differences in their measuring principals and systematic factors, where the reflectance detected by MSL was extremely close to that of the HSL system in different N levels.

### Regression analysis results among ASD, MSL, HSL reflectance and rice LNC

In SVM regression, all training and validation datasets were randomly partitioned. The descriptive statistics for 2014 and 2015 are listed in Table 1, where samples were also split by calibration and validation. Table 1 shows that the overall LNC level of Yangliangyou 6 (mean value > 19 mg/g) was higher than that of Yongyou 4949 (mean value about 13 mg/g). In addition, the large standard deviation and range of value (max – min) indicated that Yongyou 4949 had more variation in LNC than the other cultivar. This result can be attributed to the divergence in different paddy cultivars and growing locations.

**Figure 5:**
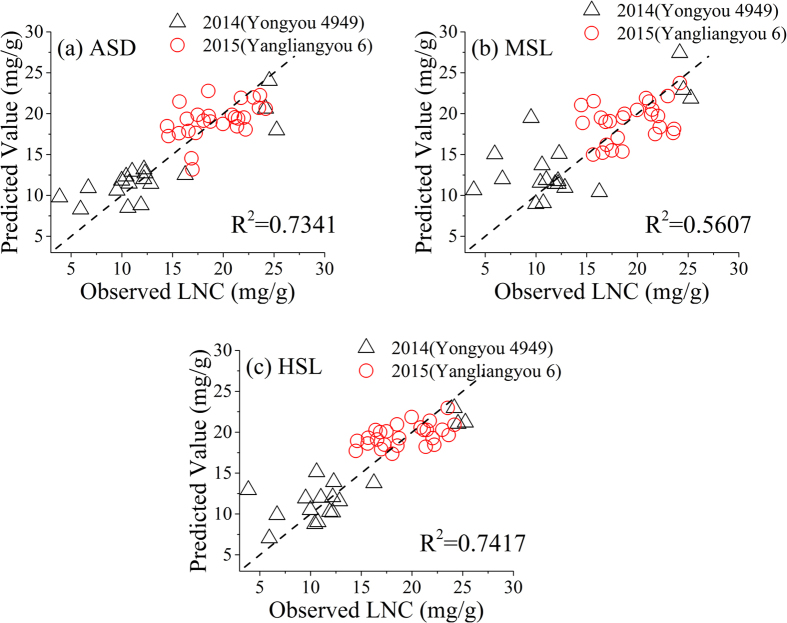
Relationship between the observed leaf nitrogen concentration (LNC) and the predicted LNC based on active and passive detectors with a support vector machine (the dashed line represents 1:1 line) based on validation dataset (n = 44) (ASD: ASD FieldSpec Pro FR (Analytical Spectral Devices, field spectroradiometer, full-range, Inc., Boulder, USA); MSL: multispectral LiDAR; HSL. hyperspectral LiDAR; N1–N3 indicateac different LNC levels).

Figure 5 shows the comparison of the linear regression results between the observed and predicted rice LNC with the three detectors. The additional spectral information improved the *R*^*2*^ from 0.56 with MSL to 0.74 with HSL (*RMSE*: 2.80 vs 3.65 mg/g, *RE*: 16.82% vs 21.94% in Table 2). The HSL system can estimate rice LNC with similar accuracy to ASD, with an *R*^*2*^ of 0.74 vs 0.73 and an *RMSE* of 2.80 vs 2.82 mg/g (Table 2). Compared with the HSL system, the ASD had more bands (601 in this study vs 32) with finer spectral resolution However, HSL is an active sensor, and had a smaller FOV (less than 3 mrad) than ASD (FOV 440 mrad). The discrepancy in reflectance curves of different N levels in Fig. 4 was more obvious with HSL than with ASD and MSL. This result suggested the sensitivity of HSL in detecting the foliar biochemical conditions compared with the two other means. Our result with HSL for rice LNC (*R*^*2*^ = 0.74) was similar to that of Eitel, *et al*.^42^, who used a dual-wavelength LiDAR to estimate the foliar nitrogen of winter wheat (*R*^*2*^ =  0.71–0.76).

The scatter of the predicted and observed rice LNC compared with the 1:1 line (Fig. 5) suggested that the accuracy of using the spectral reflectance from 400–1000 nm as predictors tended to overestimate lower rice LNC (<10 mg/g), and underestimate higher LNC (>20 mg/g), though this phenomenon was less obvious in ASD and HSL. This finding agrees with earlier findings by Eitel, *et al*.^43^ who found that the accuracy of laser-derived dry mass per unit area (*W* in t ha^−1^) estimates in winter wheat decreased when the actual *W* was large. The saturation effect described above with increasing LNC still exists even when using the complete range of reflectance instead of red light-dependent or NIR-based indices^44^. The sensitivity of reflectance signals in predicting chlorophyll concentrations can decrease in red and blue regions^45^. The diurnal changes in photosynthetic photon flux density, which affects various pigments, may be the cause for the saturation result when the plant LNC is high.

Spectral indices have been widely applied detect crop N status. For example, Eitel, *et al*.^46^ reported that an index based on ratio (MCARI/MTVI2) could best estimate LNC of winter wheat (*R*^*2*^ ranging from 0.45 to 0.69 for narrow bands). Chen, *et al*.^2^ showed that a new index named double-peak canopy nitrogen index performed best in N detection, with an *R*^*2*^ of 0.72 for corn and 0.44 for wheat. Erdle, *et al*.^44^ reported that R760/R730 was the most powerful index for detecting wheat N status. Tian, *et al*.^47^ found that R553/R537 was the best index to assess rice LNC under different conditions. The normalized difference nitrogen index (NDNI = [log (1/R_1510_) −log (1/R_1680_)]/[log (1/R_1510_) + log (1/R_1680_)]) correlated with the foliar N concentration of native shrub vegetation (*r* = 0.582, *P* = 0.004)^48^. To the best of our knowledge, no index is proved universally effective for crop LNC estimation, because of the different spatial, temporal and measurement conditions. Further research is necessary to examine the performance of HSL measurements to predict LNC under various circumstances. The observation toward crop N status is also influenced by certain factors (e.g., leaf inner structure, leaf area index), which demands a thorough and detailed study in the future.

## Conclusion

Based on a systematic analysis of the quantitative relationships between LNC and reflectance characteristics, the capability of an HSL system with 32 channels to estimate rice LNC was evaluated through comparisons with the passive hyperspectral sensor ASD FieldSpec Pro FR (using the reflectance spectra from 400 to 1000 nm) and an MSL system with four bands. Through the regression results of SVM, rice LNC was best predicted by HSL. ASD provided comparable results with HSL in this study. Limited by the number of wavelengths, the MSL provided a moderate regression.

HSL demonstrated its potential as a rapid and non-destructive tool for assessing rice LNC, which can facilitate real-time N management decisions. Additional studies should be carried out to test the monitoring relationships further using independent datasets, which can help to test the reliability under a range of conditions. In addition, the HSL system shows promise for other agronomic applications, such as examining other crop biochemical properties and soil parameters, which merits exploration in future research.

## Additional Information

**How to cite this article**: Sun, J. *et al*. Evaluation of hyperspectral LiDAR for monitoring rice leaf nitrogen by comparison with multispectral LiDAR and passive spectrometer. *Sci. Rep.* **7**, 40362; doi: 10.1038/srep40362 (2017).

**Publisher's note:** Springer Nature remains neutral with regard to jurisdictional claims in published maps and institutional affiliations.

## Figures and Tables

**Table 1:** Basic statistical information of the LNC conditions of rice samples in support vector regression in 2014 and 2015.

Season	Site	Cultivar	Dataset	Sample Size	Leaf nitrogen concentration			
					Mean	SD^a^	Min	Max
2014	Suizhou	Yongyou 4949	Training	72	13.22	6.94	4.70	35.61
			Testing	18	12.80	6.12	3.87	25.26
2015	Wuhan	Yangliangyou 6	Training	104	19.80	4.00	8.69	31.87
			Testing	26	19.26	2.97	14.46	24.18

^a^SD: standard deviation.

**Table 2:** Assessment of the support vector machine models developed with data from active (MSL: multispectral LiDAR, HSL: hyperspectral LiDAR) and passive sensors (ASD: ASD FieldSpec Pro FR) (*R* ^*2*^: coefficient of determination, *RMSE*: root mean square error, *RE*: relative error).

	*Equation*	*R*^*2*^	*RMSE (mg/g*)	*RE (%*)
ASD	y = 0.69x + 5.06	0.73	2.82	16.96
MSL	y = 0.61x + 6.97	0.56	3.65	21.94
HSL	0.47x + 10.13	0.74	2.8	16.82
